# The Effect of the Entrance of Air into the Circulation

**Published:** 1889-12-28

**Authors:** 


### RECENT RESEARCH.

### THE EFFECT OF THE ENTRANCE OF AIR INTO THE CIRCULATION.

Dr. H. A. Hare publishes in a recent *Philadelphia Therapeutic Gazette* certain experiments on the above-mentioned subject. We are taught by all text-books that the entrance of air into a vein is one of the most serious complications which may occur during an operation. But Dr. Hare shows by experiment that there is not so much danger as supposed. Upwards of seventy dogs were experimented with. The conclusions arrived at were, that enormous amounts of air must enter a vein to cause death, and that no such quantity can possibly find its way into a vein injured by the knife of the surgeon. But there are a number of cases on record in which death has been attributed to the entrance of air into veins; and death has also been attributed to the entrance of air into the uterine sinuses. It has been theorised that air produces a frothy state of the blood, which prevents the due transfer of the fluid through the pulmonary tissue, also to gaseous distension of the heart, preventing the closure of the valves. But there is no evidence to prove that air cannot go where blood may flow, and if valves can close on a current of blood there is no reason why they should not close on air. Dr. Hare thinks, as the result of his eighty experiments, that the cases recorded as death from the entrance of air into the veins really happened from other causes. Although we may not agree with all the writer advances, enough has been advanced to demand a renewal of investigations into this interesting and important subject.

